# Unmet needs and harm reduction preferences of syringe services program participants: differences by co-use of illicit opioids and methamphetamine

**DOI:** 10.1186/s12954-024-01038-2

**Published:** 2024-06-19

**Authors:** Rachel Sun, Tonazzina H. Sauda, Rachel A. Hoopsick

**Affiliations:** 1 Department of Psychology, University of Illinois Urbana-Champaign, 603 East Daniel St., Champaign, IL 61820 USA; 2 Department of Health and Kinesiology, University of Illinois Urbana-Champaign, 1206 S. Fourth St., Champaign, IL 61820 USA

**Keywords:** Injection drug use, Co-use, Unmet needs, Harm reduction preferences

## Abstract

**Background:**

The current fourth wave of the United States opioid overdose epidemic is characterized by the co-use of opioids and stimulants, including illicit opioids and methamphetamine. The co-use of these two drugs, known as “goofballing,” is associated with higher risk for several adverse outcomes, including more frequent injections, greater health risks, and higher morbidity. Considering these differences, this unique subpopulation of people who inject drugs (PWID) may also have unique unmet needs and harm reduction preferences.

**Methods:**

We collected self-reported data from participants (*N* = 50) of a syringe services program (SSP), including basic needs and harm reduction preferences. Using bivariate analyses, we examined differences between SSP participants who do and do not co-use illicit opioids and methamphetamine. Co-use was defined as reporting the use of both drugs, which may or may not have been used simultaneously.

**Results:**

In the overall sample, the mean level of need was highest for bus passes or other transportation, a person who can help you get the services you need, medication for opioid use disorder, and a job or job training. Additionally, all participants reported being either interested or very interested in fentanyl test strips, safe consumption sites, delivery of syringe service supplies, and delivery of naloxone. Those who endorsed co-use had a greater need for food, healthcare, substance use disorder treatment, a support person to help them access needed services, and bus passes or transportation.

**Conclusions:**

Unmet needs were prevalent, and the desire for more harm reduction services was high among these PWID. Results also suggest people who co-use illicit opioids and methamphetamine may have the greatest unmet needs and desire for additional harm reduction services.

## Introduction

The United States (U.S.) has previously seen three distinct waves of the opioid overdose epidemic: the first characterized by prescription opioids, the second by heroin, and the third by synthetic opioids (e.g., fentanyl) and their analogs [8]. The proliferation of fentanyl has been rapid and detrimental, accounting for a majority of all opioid-related overdoses since 2010 [15, 27]. Consequently, there has been a large and diverse public health response, in which syringe services programs (SSPs) play a critical role in combatting injection-related health risks and other substance-related harms [11, 14, 25].

Simultaneously, methamphetamine use has spread from predominantly rural regions to large swathes of the U.S., contributing to a 50-fold increase in methamphetamine-related mortality from 1999 to 2021 [21, 26]. These trends are in part reflective of an emerging fourth wave of the opioid epidemic, characterized by the combined use of methamphetamine and illicit opioids [22]. Indeed, recent findings suggest a dramatic increase in opioid and stimulant co-use mortality [10, 14, 14, 20].

There is a wealth of literature addressing the heightened risks associated with polysubstance use, including adverse mental health outcomes [23] and infectious diseases [9, 13]. More recently, studies have also examined risks specific to methamphetamine and opioid co-use. These include more frequent injecting [28], experiences of stigma [6], and considerable health risks and morbidity [16]. Given these differences, it is possible that the needs of this subpopulation are not comprehensively addressed by SSPs, which were conceptualized to provide core services primarily associated with opioid use (i.e., syringe exchange services). However, the body of literature in this area remains limited, and further investigation is warranted. This manuscript aims to assess whether, and in which domains, the needs of people who inject drugs (PWID) who report co-use of methamphetamine and opioids differ from those who report using only one of the two substances.

## Methods

The present pilot study aims to explore unmet needs and harm reduction preferences of individuals co-using methamphetamine and illicit opioids as well as PWID more broadly. Participants were recruited over a 4-month period in 2022 from a small midwestern SSP located in a metro area of fewer than 250,000 residents (classified as code 3 Metro under the Rural–Urban Continuum Codes by the U.S. Department of Agriculture). Like many regions in the U.S., the communities in and around this SSP have experienced increasing drug poisoning mortality in recent years. At the time of this study, this SSP provided needs-based syringe distribution and disposal, naloxone distribution and training, harm reduction education, infectious disease screening, and referral to other co-located services (e.g., dental care, family planning services, immunizations, food assistance). Study participants (*N* = 50) consisted of adults who had accessed syringe services and were recruited either in person or via flyers in a contact-free supplies box. After providing informed consent, eligible participants completed a brief online survey on Qualtrics, which took approximately 10–15 min to complete. The survey questions examined in the current study can be found in the appendix. Upon completion, participants received $10 in cash. The study protocol was approved by the Institutional Review Board at the University of Illinois at Urbana-Champaign.

### Measures

#### Drug use

Participants were asked the question, “In the past three months, which of the following drugs have you used? Check all that apply.” Each option included examples or colloquial names of that drug type. “Street opioids” included heroin, opium, and illicitly manufactured fentanyl. Methamphetamine included “speed,” “crystal meth,” and “ice.” Participants were considered co-users if they reported use of both illicit opioids and methamphetamine, which may or may not have been used simultaneously.

#### Unmet needs

Participants were asked to “Rate your level of need for the following supplies and services.” Items were scored as 0 (Not needed), 1 (Needed), or 2 (Urgently needed).

#### Harm reduction preferences

Participants were asked to “Rate your level of interest in the following supplies and services not currently provided by the Syringe Services Program.” Items were scored as 0 (Very uninterested), 1 (Uninterested), 2 (Neither interested nor uninterested), 3 (Interested) and 4 (Very interested).

### Analytic plan

Bivariate analyses were used to examine potential differences in the unmet needs and harm reduction preferences of PWID according to whether they reported the co-use of illicit opioids and methamphetamine or the use of just one of these substances. Correcting for multiple comparisons with the Benjamini–Hochberg procedure resulted in a *p* value cutoff of 0.017 for assessing significance [5]. All analyses were conducted with Stata version 18 (College Station, TX).

## Results

Our sample of participants ranged in age from 23 to 65 years old and consisted of men (*n* = 22), women (*n* = 27), and nonbinary people (*n* = 1). Refer to Table 1 for additional participant characteristics.

**Table 1:** Demographic characteristics of PWID accessing a midwestern syringe services program in 2022 (N = 50)

	Overall sample (*N* = 50) mean (SD) or % (n)	Use of street opioids^a^ or methamphetamine^b^ (*n* = 30) mean (SD) or % (n)	Co-use of street opioids^a^ and methamphetamine^b^ (*n* = 20) mean (SD) or % (n)	*p* value
Age, years	34.8 (8.6)	36.1 (9.6)	32.8 (6.6)	0.186
Gender identity				
Woman	54.0% (27)	60.0% (18)	50.0% (10)	0.321
Man	44.0% (22)	40.0% (12)	45.0% (9)	
Nonbinary or genderqueer	2.0% (1)	0.0% (0)	5.0% (1)	
Race and ethnicity				
Non-hispanic white	90.0% (45)	96.7% (29)	80.0% (16)	0.03
Hispanic	8.0% (4)	0.0% (0)	20.0% (4)	
More than one race	2.0% (1)	3.3% (1)	0.0% (0)	
Education				
Less than high school	16.0% (8)	16.7% (5)	15.0% (3)	0.797
High school diploma or equivalent	54.0% (27)	50.0% (15)	60.0% (12)	
At least some college	30.0% (15)	33.3% (10)	25.0% (5)	
Employment status				
Unemployed or disabled	52.0% (26)	28.0% (14)	60.0% (12)	0.024
Working part-time	32.0% (16)	26.7% (8)	40.0% (8)	
Working full-time	16.0% (8)	26.7% (8)	0.0% (0)	
Income				
Less than $10,000	40.0% (20)	30.0% (9)	55.0% (11)	0.056
$10,000–$29,999	38.0% (19)	40.0% (12)	35.0% (7)	
$30,000–$49,999	16.0% (8)	26.7%% (8)	0.0% (0)	
$50,000 or more	6.0% (3)	3.3% (1)	10.0% (2)	
Living situation				
In a house or apartment	72% (36)	73.3% (22)	70.0% (14)	0.056
In my car, unsheltered on the street, under a bridge, etc	16% (8)	6.7% (2)	5.0% (1)	
Motel or hotel	6.0% (3)	0.0% (0)	10.0% (2)	
Other	6.0% (3)	12.0% (6)	15.0% (3)	
Drug use				
Illicit opioids	82.0% (41)	–	–	
Methamphetamine	58.0% (29)	–	–	
Cannabis	54.0% (27)	–	–	
Sedatives	36.0% (18)	–	–	
Cocaine	36.0% (18)	–	–	
Prescription opioids	28.0% (14)	–	–	
Other	20.0% (10)	–	–	

^a^Illicit opioids included heroin, opium, and illicitly manufactured fentanyl

^b^Methamphetamine included “speed,” “crystal meth,” and “ice

In the overall sample, the mean (± SD) level of need was highest for bus passes or other transportation (1.48 (± 0.71)). Other needed items include a person who can help you get the services you need (1.26 (± 0.72)), medication for opioid use disorder (1.08 (± 0.72)), and a job or job training (1.02 (± 0.82)). Additionally, all participants reported being either interested or very interested in fentanyl test strips, safe consumption sites, delivery of syringe services supplies, and delivery of naloxone. Responses to other items are shown in Table 2.

**Table 2:** Level of need or preference for supplies and services reported by PWID accessing a midwestern syringe services program in 2022

	Overall sample (*N* = 50) mean (SD)	Use of illicit opioids^a^ or methamphetamine^b^ (*n* = 30) mean (SD)	Co-use of illicit opioids^a^ and methamphetamine^b^ (*n* = 20) mean (SD)	*p* value
Needs				
Housing or shelter	0.98 (0.87)	0.77 (0.86)	1.30 (0.80)	0.032
Food	0.88 (0.75)	0.63 (0.72)	1.25 (0.64)	**0**.**003**
Job or job training	1.02 (0.82)	0.83 (0.87)	1.30 (0.66)	0.048
Healthcare	0.84 (0.62)	0.67 (0.61)	1.10 (0.55)	**0**.**014**
Testing and treatment for HIV	0.52 (0.61)	0.40 (0.56)	0.70 (0.66)	0.091
Testing and treatment for hepatitis C	0.86 (0.73)	0.70 (0.70)	1.10 (0.72)	0.056
Medication for opioid use disorder	1.08 (0.72)	0.90 (0.66)	1.35 (0.75)	0.03
Substance use disorder treatment	1.04 (0.64)	0.87 (0.57)	1.30 (0.66)	**0**.**017**
Mental health treatment	0.96 (0.73)	0.97 (0.67)	0.95 (0.83)	0.938
A person that can help you get the services you need	1.26 (0.72)	1.00 (0.74)	1.65 (0.49)	**0**.**001**
Bus passes or other transportation	1.48 (0.71)	1.23 (0.73)	1.85 (0.49)	**0**.**001**
Wound treatment	0.58 (0.70)	0.47 (0.68)	0.75 (0.72)	0.165
Other safer injection supplies	0.90 (0.65)	0.77 (0.68)	1.10 (0.55)	0.074
Preferences				
Fentanyl test strips	3.58 (0.78)	3.50 (0.90)	3.70 (0.57)	0.383
Safer smoking supplies	2.74 (1.12)	2.63 (1.22)	2.90 (0.97)	0.416
Safe consumption site	3.18 (0.85)	3.13 (0.94)	3.25 (0.72)	0.639
Delivery of syringe services supplies	3.64 (0.75)	3.57 (0.90)	3.75 (0.44)	0.402
Delivery of naloxone	3.44 (0.93)	3.23 (1.10)	3.75 (0.44)	0.053

T-tests for differences in means. Bold values are statistically significant after Benjamini–Hochberg correction

^a^Illicit opioids included heroin, opium, and illicitly manufactured fentanyl

^b^Methamphetamine included “speed,” “crystal meth,” and “ice.”

Analyses revealed significant differences between participants who did and did not endorse co-use of illicit opioids and methamphetamine. From our unmet needs measure, we observed significantly greater need among co-users of food (*p* = 0.003), healthcare (*p* = 0.014), substance use disorder treatment (*p* = 0.017), and a person that can help them get the services they need (*p* = 0.001). Responses to our harm reduction preferences measure did not differ by co-use status, however this could be due to a ceiling effect since all participants reported a high level of interest in all items.

## Discussion

Among this sample of PWID, unmet needs were prevalent and desire for more harm reduction services was high, especially among those who co-use methamphetamine and illicit opioids. The current study is consistent with previous work identifying mental health services, housing, harm reduction, and infectious disease prevention as critical for PWID [18]. Unsurprisingly, fentanyl strips were also identified as a critical need, reflecting the high mortality risk associated with synthetic opioids [7, 30]. In addition, our results add to the small but growing body of literature examining methamphetamine and illicit opioid co-use by extensively assessing co-users for a broad range of needs and preferences.

While we observed a moderate level of need for many healthcare-related supplies and services, (e.g., treatment for Hepatitis C, injection supplies, mental health treatment), many of the most critical areas of need identified by PWID lie beyond healthcare. Most participants in the current study reported urgently needing a job or job training, bus passes or other transportation, or food; these needs were amplified among co-using participants. However, these basic living necessities are beyond the scope of services offered by most SSPs.

As SSPs do not holistically address all of the needs of PWID, alternative models of care may be better positioned to provide or connect individuals to certain resources. Peer recovery support services (PRSS), for instance, have been found to reduce substance use disorder (SUD) relapse rates, improve social support, and increase treatment retention rates [12]. These groups are led by individuals with lived experience of SUDs who provide a range of support including transportation to healthcare appointments, assistance with insurance enrollment, and connecting participants to other resources [24]. As such, they may be particularly beneficial for co-using individuals who have greater needs for basic living necessities, are less likely to be enrolled in treatment, and find drug use to be more central to their identity [6]. Moreover, research suggests that PRSS are well-positioned to incorporate harm reduction services, and these hybrid models have been shown to be a feasible way to deliver a broader range of services to PWID and engage populations that are often underserved [3, 4]. Additionally, an expansion of services offered by SSPs to include more resources and services (e.g., food assistance, Medicaid enrollment, on-site primary care services) could better address the needs and preferences of PWID.

Finally, while the needs of PWID are manifold, their ability to access vital services are limited [2]. In our study, a person who can help PWID get the services they need was among the most urgent needs, especially among co-using participants. Indeed, the structure of many public health interventions (e.g., sustained treatment models) often collide with the lived experiences of PWID [29]. Many qualitative studies have emphasized that for PWID, immediate priorities (e.g., food, shelter) and existing difficulties impede treatment accessibility [1].

In conclusion, we observe a critical need for both basic living and health-related supplies and services. Moreover, we found high levels of interest in delivery services, fentanyl test strips, and safe consumption sites. Participants who endorsed the co-use of illicit opioids and methamphetamine reported significantly greater levels of need for numerous basic living necessities, social services, and substance use disorder treatment. Following these findings, we encourage SSPs in the community we surveyed to include or link to broader support services, such as PRSSs, to address underlying social needs. Further research with other SSPs in other settings is needed to confirm if our findings are generalizable to the needs and preferences of PWID, more broadly. Ultimately, continued efforts to expand accessibility, legality, and breadth of services providing comprehensive prevention, harm reduction, and healthcare services for PWID are vital.

### Limitations

Sample size is a major limitation of the current pilot study. Our study was exploratory in nature, and further investigation is necessary to confirm our findings or uncover other differences. Future studies should examine potential differences in harm reduction behaviors and harm reduction self-efficacy of PWID by co-use status, given that PWID who co-use illicit opioids and methamphetamine appear to have substantially more unmet needs compared to those who do not co-use. Moreover, qualitative research could supplement the findings of the current study and may further elucidate the unmet needs and harm reduction preferences of PWID. A second limitation is the lack of racial and ethnic diversity in our sample, therefore limiting this study’s ability to capture potentially unique characteristics of non-white PWID. Although non-white PWID are highly represented in total opioid overdose deaths, feelings of shame, mistrust for predominantly white institutions, and an increased fear of policing continue to drive disparities in SSP access [17]. Finally, we did not assess whether participants who reported use of both methamphetamine and illicit opioids also used the two simultaneously. Simultaneous use (sometimes referred to as “goofballing”) produces greater effects than using either drug alone [19], and is associated with additional risks, including homelessness, injecting daily, and self-reported opioid overdose [13, 16, 25]. However, it is unknown whether the increased needs of co-using PWID in this study were associated with “goofballing.”

### Conclusions

Findings from the current pilot study suggest that PWID have many unmet needs and desire additional harm reduction services. Our exploratory analyses also suggest that people who co-use illicit opioids and methamphetamine may have the greatest unmet needs and desire for additional harm reduction services. There is an imminent need for expanded access to a wider breadth and depth of harm reduction services for PWID in the U.S., particularly for those who co-use illicit opioids and methamphetamine.

## Data Availability

Data will be provided upon reasonable request by the corresponding author.

## Appendix. Survey items assessed in the current study

What is your age in years? _____

What is your gender?

- Man
- Woman
- Non-binary, genderqueer, or other gender

How would you describe yourself? Check all that apply.

- American Indian or Alaska Native
- Asian
- Black or African American
- Hispanic or Latino
- Native Hawaiian or Other Pacific Islander
- White
- Other, please specify: _____________________

What is the highest degree or level of school you have completed?

- Less than high school
- High school diploma or equivalent (GED)
- Trade school
- Some college (no degree)
- Completed Associate’s or other Technical 2-year degree program
- Completed Bachelor’s degree or other 4-year degree program
- Some Graduate or Professional studies (completed 4-year degree but not Graduate degree)
- Completed Graduate or Professional degree (Master’s degree or higher)
- Other, please specify: _____________________

Which of the following best describes your employment situation?

- Working full-time
- Working part-time
- Unemployed and looking for work
- Disabled
- Retired
- Taking classes/in an unpaid training program
- Other, please specify: ______________________

What was your TOTAL HOUSEHOLD income in the last 12 months?

- Less than $10,000
- $10,000–$19,999
- $20,000–$29,999
- $30,000–$39,999
- $40,000–$49,999
- $50,000–$74,999
- $75,000 or more

Where did you sleep last night?

- In a house or apartment I own or rent
- In house or apartment owned or rented by someone else
- In an institutional setting (including hospital, jail, prison, juvenile detention facility, long-term care facility, or nursing home)
- In an emergency shelter, safe haven, or transitional housing project
- Motel or hotel
- In my car, unsheltered on the street or under a bridge, etc
- Other, please specify: ______________________

In the past three months, which of the following drugs have you used?

- Prescription stimulants (Ritalin, Concerta, Dexedrine, Adderall, diet pills, etc.)
- Prescription sedatives or sleeping pills (Valium, Serepax, Ativan, Xanax, Librium, Rohypnol, GHB, etc.)
- Prescription opioids (fentanyl, oxycodone, Oxycontin, Percocet, hydrocodone, Vicodin, methadone, Lortab, buprenorphine, etc.)
- Cannabis (marijuana, pot, grass, hash, etc.)
- Cocaine (coke, crack, etc.)
- Methamphetamine (speed, crystal meth, ice, etc.)
- Inhalants (nitrous oxide, glue, gas, paint thinner, etc.)
- Hallucinogens (LSD, acid, mushrooms, PCP, Special K, ecstasy, etc.)
- Street Opioids (heroin, opium, street fentanyl, etc.)
- Other, please specify:__________________________________

Rate your level of need for the following supplies and services:

**Table:**

	Not needed	Needed	Urgently needed
Housing/shelter			
Food			
Job or job training			
Healthcare			
Testing and treatment for HIV			
Testing and treatment for hepatitis C			
Medication for opioid use disorder (suboxone, vivitrol, buprenorphine)			
Substance use disorder treatment			
A person that can help you get the services you need			
Bus passes or other transportation			
Fentanyl test strips			
Wound treatment			
Other safer injection supplies			
Safer smoking supplies (e.g., glass pipes, foil, copper wire filters)			

Rate your level of interest in the following supplies and services not currently provided by [Name of Syringe Services Program]:

**Table:**

	Very interested	Interested	Neither interest nor uninterested	Uninterested	Very uninterested
Fentanyl test strips (can tell you if your drugs contain fentanyl)					
Safer smoking supplies (e.g., glass pipes, foil, copper wire filters)					
Safe consumption site (also known as a supervised injection facility or overdose prevention site)					
Delivery of syringe service supplies					
Delivery of Naloxone (Narcan)					
